# Importance of Zika Virus NS5 Protein for Viral Replication

**DOI:** 10.3390/pathogens8040169

**Published:** 2019-09-30

**Authors:** Hesham Elshahawi, Sharifah Syed Hassan, Vinod Balasubramaniam

**Affiliations:** 1 Jeffrey Cheah School of Medicine and Health Sciences, Monash University Malaysia, Jalan Lagoon Selatan, Subang Jaya 47500, Selangor, Malaysia; elshahawi.hesham@gmail.com (H.E.); Sharifah.SyedHassan@monash.edu (S.S.H.); 2 Tropical Medicine & Biology Multidisciplinary Platform, Monash University Malaysia, Jalan Lagoon Selatan, Subang Jaya 47500, Selangor, Malaysia

**Keywords:** Zika, NS5, Flavivirus, Arbovirus, TBK1, STAT2, IFN1, IFN3, RdRp, MTase

## Abstract

Zika virus is the latest addition to an ever-growing list of arboviruses that are causing outbreaks with serious consequences. A few mild cases were recorded between 1960 and 1980 until the first major outbreak in 2007 on Yap Island. This was followed by more severe outbreaks in French Polynesia (2013) and Brazil (2015), which significantly increased both *Guillain-Barre* syndrome and microcephaly cases. No current vaccines or treatments are available, however, recent studies have taken interest in the NS5 protein which encodes both the viral methyltransferase and RNA-dependent RNA polymerase. This makes it important for viral replication alongside other important functions such as inhibiting the innate immune system thus ensuring virus survival and replication. Structural studies can help design inhibitors, while biochemical studies can help understand the various mechanisms utilized by NS5 thus counteracting them might inhibit or abolish the viral infection. Drug repurposing targeting the NS5 protein has also proven to be an effective tool since hundreds of thousands of compounds can be screened therefore saving time and resources, moreover information on these compounds might already be available especially if they are used to treat other ailments.

## 1. Introduction

**Figure 1:**
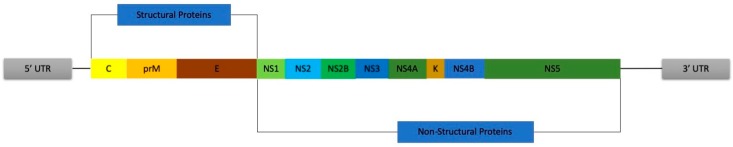
Zika virus (ZIKV) single stranded positive RNA with its single open reading frame (ORF) flanked by the 5′ and 3′ untranslated regions (UTRs). The regions coding for the structural and non-structural proteins are shown, as well as the individual proteins.

Zika virus (ZIKV) is a single stranded positive sense RNA arbovirus belonging to the *Flaviviridae* family alongside Dengue virus (DENV), Yellow fever virus (YFV), and West Nile virus (WNV) [1]. The 11kb RNA strand has a single open reading frame (ORF) that is flanked by a 5′ and 3′ untranslated regions (UTRs) that assist in translation (refer to Figure 1). The ORF codes a single polyprotein that is processed and cut to yield 3 structural proteins: C→ capsid, prM→ pre-membrane, E→ envelope and 7 non-structural proteins: NS1, NS2A, NS2B, NS3, NS4A, NS4B and NS5 [2,3]. Similar to other arboviruses, ZIKV infects humans mainly via the bite of an infected female *Aedes* mosquito while it can be also maintained in sylvatic cycles (transmission between mosquitoes and wild animals) [4,5,6,7]. The virus incubates for 5–10 days in the mosquito’s midgut before invading the salivary glands thus infecting its next host as the mosquito feeds [8,9]. Finally, non-vector transmission routes have also been recorded, this includes sexual and vertical transmission (between mother and fetus) alongside various bodily fluids [10,11,12]. ZIKV was first isolated from rhesus monkey 766 in the Zika forest in Uganda in 1947 as part of a yellow fever surveillance study [13]. This was followed by the first recorded human infections in 1952 in Uganda and Tanzania [14,15,16]. There were 14 sporadic cases reported between the 1960s and 80s without serious consequences since the disease presented as a self-limiting febrile illness [17,18]. The year 2007 marked the first ZIKV outbreak and spread outside of Africa and Asia with 49 confirmed and 59 suspected ZIKV cases being reported on Yap Island. Duffy et al. estimated that approximately 3/4 of the population above 3 years of age showed serological evidence of a recent ZIKV infection [18]. This was followed by a second outbreak in French Polynesia in 2013 which swept across the Pacific affecting multiple islands including Cook Islands, New Caledonia, Fiji, Samoa, the Solomon Islands and Easter Island before finally reaching the Americas by 2015 as predicted [16,18,19].

The 2013 outbreak led to an increased interest in ZIKV since this was the first time the virus was linked to hospitalisation and neurological complications. Recorded cases in French Polynesia only affected 3% of the population but led to hospitalisation and a 20-fold increase in reported cases of *Guillain-Barre* syndrome (GBS) [20]. Brazil suffered heavily by the epidemic since there was a 20-fold increase in microcephaly and approximately 1.3 million reported cases of autochthonous infection [21]. Finally, the outbreak reached the USA by 2015 [22] and started declining worldwide by the end of 2016 without any signs of resurgence [23].

## 2. NS5

### 2.1. Protein Structure

The non-structural protein 5 (NS5), is the largest product coded by the ZIKV RNA being around 904 amino-acids long [24,25]. Zika virus NS5 consists of 2 domains that enhance each other’s functions; an RNA-dependent RNA polymerase (RdRp) domain at the C-terminal connected via a linker to a methyltransferase (MTase) domain at the N-terminal. The NS5 protein is important for viral replication, survival and immune system evasion alongside other roles [26,27,28,29,30]. Zhao et al. found that the ZIKV NS5 protein shares a lot of structural similarities with the Japanese encephalitis virus (JEV) due to conserved residues in the loops and beta sheets forming the MTase domain [30].

### 2.2. Viral Replication

Potisopon et al. found that both the RdRp and MTase domains interact with one another to increase the efficiency of RNA replication in DENV. The first stage involves synthesising primers complementary to the 3′ end of the genome followed by a conformational change in a transition phase to be able to elongate the RNA. Experiments comparing the kinetic of each phase between a recombinant NS5 and the RdRp domain showed that the MTase domain is required to increase the efficiency of initiation, priming and elongation (6–17 folds higher). A similar outcome was observed in ZIKV NS5 by Zhao et al. and the same can be said for other flavivirus NS5 proteins [28,30].

The NS5 MTase domain has been observed by Issur et al. to work alongside NS3 to form the virus’ capping mechanism; NS5 is a true guanylyl-transferase, while NS3 acts as the RNA triphosphatase (RTase) in the reaction [25]. This is important in preventing the newly formed mRNA from being detected by the innate immune system while also ensuring its translation by the host translation machinery [31]. The first step involves the hydrolyses of adenosine nucleotide in the initiating position at the 5′ end of the RNA by the NS3 RTase. Following that, the NS5 MTase forms an enzyme-substrate complex with a GMP molecule which is followed by transferring the GMP to the 5′ diphosphate end of the RNA and finally the methylation of the cap at the 2nd OH (2′OMTase activity by NS5) using S-Adenosyl methionine (SAM), as a methyl donor. Furthermore, a 6-fold allosteric enhancement of the NS5 reaction via binding to NS3 at the linker between the RdRp and MTase domains was observed [25].

## 3. ZIKV and the Innate Immunity

### 3.1. Activation of IFN Signalling

After viral infection, the body relies on its innate immune system to hold the infection at bay, while the adaptive immune system can launch a more effective response. This is achieved via the secretion of various cytokines which are downstream targets of the interferon signalling pathway [32]. There are 3 signalling pathways that are activated by interferons (IFN) type I (α and β), type II (γ) and type III (λ) [33,34]. IFN type I and IFN type III signalling have been shown to exert strong antiviral effects and to be antagonised by ZIKV NS5 whereas IFN type II signalling has been shown to be involved in ZIKV infection [35].

**Figure 2:**
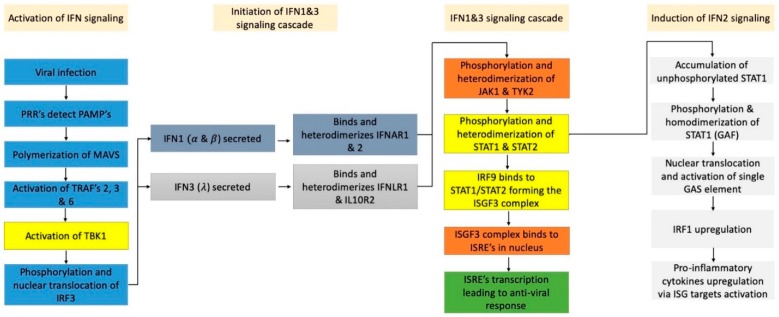
Cascade of events that leads to interferons (IFN) mediated antiviral response. The blue pathway leads to the phosphorylation and nuclear translocation of IRF3 thus activating the transcription of the IFN1 and IFN3 genes. This allows the secretion of both IFNs followed by the binding to their respective receptors leading to the activation of the JAK1/TYK2 pathway (depicted in orange). This is followed by the formation of the tripartite transcription factor (ISGF3) complex thus activating interferon stimulated response elements (ISRE) sites resulting in an effective antiviral response (depicted in green). After STAT2 degradation, STAT1 phosphorylation and homo-dimerization increases leading to the formation of gamma activated factor (GAF) elements and up-regulation of IRF1 [44]. This leads to the up-regulation of pro-inflammatory cytokines. ZIKV NS5 interferes at the steps colored in yellow; NS5 binds to TBK1 preventing its phosphorylation and activation by the TRAF’s and also binds STAT2 marking it for proteasomal degradation [26,27,45].

Following cellular entry, ZIKV releases its RNA followed by the assembly of replication factories at the evaginations of the endoplasmic reticulum. The newly synthesised RNA has a 5′-triphosphorylated end which is an example of a pathogen associated molecular pattern (PAMP). Retinoic acid-inducible gene I (RIG-1) and melanoma differentiation-associated gene 5 (MDA5) are important pathogen recognition receptors (PRRs) which detect the nascent 5′-triphosphorylated RNA and activate the mitochondrial antiviral signalling (MAVS) protein (refer Figure 2). Once MAVS is active, a downstream signalling cascade recruits tumor necrosis factor (TNF) receptor associated factors (TRAF’s) 2, 3 and 6 leading to the activation of TANK-binding Kinase 1 (TBK1) thus phosphorylating and translocating interferon regulatory factors 3 and 7 (IRF3 and IRF7) to the nucleus to activate the transcription of IFN type I and IFN type III [36,37,38,39,40].

Both IFN type I and type III act directly by massing an antiviral response while stimulating the adaptive immune system to design antibodies targeting ZIKV. As seen in Figure 2, type I and type III INFs bind to different receptors; IFNAR1 and IFNAR2 for IFN α and β whereas IFN type III binds to the IFNLR1 and IL10R2 receptors. Each receptor pair forms a heterodimer on the cell surface and once activated, both receptor pairs follow a similar downstream signalling cascade as seen in Figure 2. This involves recruiting, phosphorylating and the hetero-dimerization of janus kinase 1 (JAK1) and tyrosine kinase 2 (TYK2) leading to the phosphorylation and hetero-dimerization of signal transducer and activator of transcription 1 (STAT1) and (STAT2). IRF9 binds to the STAT1/STAT2 heterodimer forming the ISGF3 complex which migrates to the nucleus and binds to various interferon stimulated response elements (ISREs), thus transcribing various interferon stimulated genes (ISGs) that deal with the ZIKV infection [41,42]. Zhou et al. conducted a gene array study to determine if both IFN type I and IFN type III activated different genes but found that all of IFN type III’s targets are also activated by IFN type I [43].

### 3.2. NS5 Antagonism of IFN1 and IFN3 Signalling

Kumar et al. studied the various effects of ZIKV infection on the innate immune system. Their first observation was ZIKV’s ability to abolish the host cell’s IFN response and signalling by measuring the levels of interferon beta (IFNb) and interferon induced protein with tetratricopeptide repeats 1 (IFIT1) using quantitative real-time PCR (qPCR). They noticed that the peak levels were only attained between 24 and 48 h unlike the control’s 12 h. Hertzog et al. attributed this to the NS5 protein; NS5 binds and marks STAT2 for proteasome-dependent degradation, while also lowering STAT1 phosphorylation levels. Furthermore, lower activation levels of the IFIT1 promoter which is both, an ISRE and an IRF3 target shows that the virus is antagonizing IFN type I and III signalling at both the induction and effector phases [27,40].

Lin et al. suggested a mechanism by which ZIKV NS5 abolishes the IFN response via an upstream pathway; this involves antagonizing IRF3 thus preventing the transcription of IFN1 and IFN3 genes (refer to Figure 2). This is attained by preventing the phosphorylation and nuclear translocation of IRF3; IRF3 levels remained constant after infection unlike the 70% decrease in phosphorylated IRF3. ZIKV NS5 is able to do this by binding to TBK1 via its ubiquitin-like domain (ULD), both the MTase and RdRp domains are required as the absence of one showed no decrease in phosphorylated IRF3. Furthermore, TBK1 binds to the groove formed by the MTase/RdRp linker and forming this complex might interfere with TRAF 6′s interaction and binding with the TBK1 C-terminal scaffolding/dimerization domain (SDD) due to their close proximity. Finally, this leads to the inability to activate TBK1 which in turn cannot phosphorylate, and activate IRF3 thus abolishing type I and III interferon transcription [45].

### 3.3. NS5 Causes the Degradation of STAT2

Grant et al. studied the STAT2 degradation mechanism and found that it slightly differs from the mechanism utilised by DENV; ZIKV NS5 does not recruit the E3 ubiquitin ligase UBR4. Furthermore, they concluded that ZIKV NS5 stays in the nucleus until cytosol STAT2 levels increase after which the NS5 exits the nucleus to bind to and degrade the STAT2 thus preventing it from forming the ISGF3 complex and activating ISRE targets. This effect was reversed with the treatment of proteasome inhibitors. In addition to that, NS5 uses its MTase domain to bind to STAT2 preparing it for degradation but requires the full length NS5 to complete the process [26,27]. Finally, ZIKV NS5 only interacts with human and other non-human primate STAT2 and while wild type mice are immune, IFN deficient mice were susceptible to ZIKV infection, thus showing the importance of this signalling pathway in fighting infection [26,46].

### 3.4. Selective Induction of Type II IFN Signalling

Type II IFN signalling is also utilised in antiviral activities by transcribing a different subgroup of interferon stimulated genes (ISGs) that are pro-inflammatory [40]. Once type II IFN activates the interferon gamma receptor (IFNGR1) and (IFNGR2) receptors, JAK 1 and JAK2 are recruited and phosphorylated to prime the IFNG1 and IFNGR2 complex for the phosphorylation and homodimerization of STAT1. This forms the gamma activated factor (GAF) which is translocated into the nucleus to transcribe the single interferon gamma activated site (GAS) element IRF1 [44]. IRF1 targets certain ISG’s which transcribe pro-inflammatory cytokines like CXCL10 and also the up-regulation of the ZIKV entry factors AXL, Tyro3 and DC-SIGN [47,48]. The above pathway can be upregulated thus overexpressing IRF1. This is possible without the actual secretion of IFN2 (refer to Figure 2) since there is an accumulation of STAT1 in the cytoplasm due to STAT2 degradation via ZIKV NS5; there is not enough STAT2 to dimerize with STAT1 therefore more STAT1 homodimers will form. This leads to increased inflammation, autophagy and a spike in programmed cellular death thus spreading viral particles to infect nearby cells [35,49].

## 4. ZIKV NS5 as An Antiviral Target

As seen above, the NS5 protein has various important roles in ensuring the virus replication and survival thus making it an interesting candidate for antiviral targeting [26,27,35,40,41,45,50]. We have discussed below some of the methods that might prove useful in the pursuit of effective antivirals against Zika NS5 protein.

### 4.1. Structural Studies

Various groups have studied and released the crystalized structures of both the RdRp and MTase domains alongside identifying important and conserved residues. It has been noted that ZIKV NS5 shares a lot of similarities with other flaviviruses such as the 70 % homology of its RdRp with both WNV and JEV, the ability of its thumb, palm and fingers domains to superimpose on those of DENV 2 and 3 alongside the 76% and 81% similarity of their priming loops respectively. In addition to that, the ZIKV MTase domain was crystalized into a homodimer with both promoters folding in a similar way to DENV 3 and WNV. DENV RNA capping analogues, S-adenosylmethionine (SAM) analogues and allosteric inhibitors were found to be more effective at inhibiting the ZIKV MTase. Furthermore, the specificity of SAM analogues can be improved by either utilising the hydrophobic cavity adjoining the SAM binding site which is conserved among flaviviruses or exploiting the positively charged RNA binding tunnel [30,51,52,53,54].

Duan et al. studied the structures and inhibitors of DENV and ZIKV NS5 and found huge similarities including conserved residues among both viruses. This was confirmed when they found that almost all the involved residues in the binding of each domain with known DENV antivirals were conserved. This included the MTase inhibitor compound 10*, the RdRp inhibitor NTD107 and the N pocket inhibitors JJ-31-MG46 and compound 2 [53].

Another study found that the pyridoxine-derived, small molecule, non-nucleoside inhibitor (NNI) DMB213 inhibited the ZIKV NS5 RdRp domain with an IC_50_ of 5.2µM. The compound DMB213 was originally designed to chelate the divalent metal ions from the active sites of viruses, thus making it a direct competitor to the NS5 RdRp’s substrate. Docking analysis revealed that DMB213 sits within close proximity to the active site residues D536, D666 and D667 which are known to coordinate the Mg^2+^ ions thus confirming the chelation mechanism. Finally, the S604T mutation that renders the RdRp immune to Sofosbuvir has no effect on DMB213 thus providing an alternative option [55].

Lin et al. screened an anti-infection food and drug agency USA (FDA) approved compound library and found a potential ZIKV NS5 RdRp inhibitor. Interestingly, 10-undecenoic acid zinc salt (UA) which is currently an over the counter anti-fungal and antiviral cream was found to be a strong, direct, NNI of the RdRp domain with an IC_50_ of 1.13-1.25 µM. Docking analysis revealed that UA coordinates with residues D535, D665 and D666 at the active site of NS5 but only interacted with residues D535 and C667 via a hydrogen and a covalent bond respectively. Furthermore, residue D535 was mutated to A535 while residue D692 (outside the active site) was mutated to A692. This led to the weak binding of UA to the RdRp since A535′s IC_50_ was 20-fold higher while A692′s IC_50_ remained unchanged compared to the wild type RdRp thus proving that residue D535 is critical for UA binding [56].

### 4.2. Biochemical Studies

Rusanov et al. observed a key interaction between the ZIKV NS5 MTase and RdRp domains that affects RNA elongation. Residues E112, P113 and L115 from the MTase domain interact with residue F466 on the RdRp domain to stabilise the F motif. Mutations introduced at these residues significantly lowered RNA elongation but had no effect on initiation. Motif F forms the top part of the nucleoside triphosphate entry tunnel and the above residue interactions ensure its correct orientation. Furthermore, this feature seems to be virus specific due to differences in the arrangement of the RdRp sub-domains but drugs can be designed to interfere with this interaction thus inhibiting viral replication [57,58,59].

The NS3 helicase has an important function in ZIKV replication as it is responsible for unwinding the newly synthesised dsRNA. Due to its low efficiency after unwinding 18 base pairs, there has to be a mechanism that enables it to unwind the 11kb genome. Xu et al. observed an interaction between NS3 and NS5 that increases NS3′s dsRNA unwinding capability and found that abolishing this interaction via mutations in the NS3 C-terminal was sufficient to disrupt ZIKV replication [60]. In addition to that, it is safe to assume that drugs designed to target the NS3 C-terminal residues 303-618 and the RdRp domain residues 320-368 [61] can abolish this interaction therefore inhibiting ZIKV.

Kovanich et al. recently conducted a ZIKV and JEV interactome study that identified 137 human proteins that interact with NS5 (81 were novel). They also constructed a protein-protein interaction map consisting of 115 unique proteins with a total of 421 interactions, most of which were accumulated within spliceosome related proteins. Furthermore, flavivirus NS5 was shown to interact with some of the proteins that make up the Paf1 complex. It was found that inhibition of this complex increased infectivity of ZIKV and DENV but inhibited JEV thus showing that some host proteins interact differently with different flaviviruses [62]. Finally, NS5 is highly conserved among different flaviviruses [26,50,53,63], so identifying possible host protein interactions might prove an effective way to develop antivirals.

### 4.3. Drug Repurposing

Another interesting avenue looking at anti-viral against NS5 of Zika involves drug repurposing. Hundreds of thousands of drugs can be screened against ZIKV NS5 to test for inhibition. This includes antivirals, antibiotics, anti-cancer and anti-parasitic. Time and resources can be saved since efficacy and safety reports already exists.

One approach is to screen current nucleoside analogues since they have shown their effectiveness in viral inhibition since the 1980′s [64]. These are either purine or pyrimidine analogues which mimic their structure to trick the viral RNA/DNA polymerase into incorporating them into the growing chain thus leading to early termination. Other desirable attributes include efficient uptake and activation inside the target cells. Finally, nucleoside analogues have shown a higher affinity towards viral polymerases which might be due to the extensive error-correction mechanisms employed by mammalian cells [65,66].

Sofosbuvir is one of 7 FDA approved NS5 inhibitors that was originally designed to treat Hep C (a flavivirus). It has successfully shown inhibition towards ZIKV NS5 [63,67,68]. This can be attributed to the highly conserved sequence of the flavivirus RdRp that interacts with Sofosbuvir. Sacrametno et al. reported that Sofosbuvir successfully lowered ZIKV mediated cell death and infectivity in hepatoma, neuroblastoma, neural stem cells and brain organoids. This was achieved by Sofosbuvir’s ability to disrupt the hydrogen bonding network inside the RdRp’s active site thus leading to early chain termination. In addition, significantly higher A to G mutations were also recorded [63,69,70].

On the same note, 7DMA; another nucleoside analogue that was originally designed for Hep C treatment [71] was also found to inhibit other flaviviruses; specifically, TBEV and DENV with EC_50_ values ranging from 5 µM to 15 µM [72,73]. Eyer et al. screened 29 nucleoside analogues for anti ZIKV properties and observed that 7DMA was effective against ZIKV with an EC_50_ of 8.92 µM. In addition to that, the following 2′-C-methylated nucleoside analogues were also effective against ZIKV: 2′-CMA, 2′-CMC, 2′-CMG and 2′-CMU with EC_50′_s of 5.26 µM, 10.51 µM, 2.25 µM and 45.4 µM respectively. The above 5 compounds were the only ones that exhibited anti ZIKV properties [66], this is in line with the findings of Hercík et al. on the importance of the 2′-C-methylation of nucleoside analogues in the inhibition of NS5 RdRp [74]. Furthermore, no cytotoxicity was observed except with the 2′-CMC analogue which caused a 30% reduction in cell viability at 100 µM exposure [66]. In addition to that, Zmurko et al. found that 7DMA treatment can be tolerated by AG129 mice while significantly lowering plasma viral loads 3-8 days post ZIKV infection thus delaying morbidity and death [75].

Galidesivir, also known as BCX4430 is another nucleoside analogue with antiviral capabilities spanning 8 virus families including Rift Valley fever virus from the *Phenuiviridae* family and Ebola from the *Filoviridae* family while also being well tolerated by monkey and rodent models [76,77]. It was found to significantly lower ZIKV loads in plasma, saliva, urine and cerebral spinal fluid (CSF) samples in rhesus macaques. Macaques treated within 24 h of infection either showed low viral loads or none at all while delayed treatment of up to 72 h offered partial protection with lower plasma viral loads compared to the controls [78]. In addition to that, 7 out of 8 ZIKV infected AG129 mice treated with 300mg/kg (high dose) of BCX4430 daily survived unlike all 16 controls which had a median survival of 15.5 days. Mice given a low treatment dose of 150mg/kg/day showed signs of disease after 23 days thus showing BCX4430′s ability to delay morbidity onset. Finally, a 24-h delay in treatment did not affect survival rates while a 5-day delay still significantly delayed mortality [79].

## 5. Conclusions

ZIKV was not an important human pathogen until the 2007 and 2013 outbreaks when it was linked to GBS and microcephaly. With no assurances of a repeated outbreak not happening in the future and the lack of current treatments, anti-virals need to be developed soon. NS5 has proven in multiple studies its importance for both the survival and infectivity of ZIKV thus making it a prime target for drug development. With its crystalized structure published by multiple groups, antivirals can be designed to either inhibit the RdRp domain or target the various allosteric interactions between NS5 and its cofactors. Another approach involves repurposing current drugs especially those that show anti-NS5 potential in other flaviviruses due to the highly conservative nature of the protein. Furthermore, more interactome studies would prove useful as they would help us better understand which host proteins to target causing maximum inhibition to ZIKV. Finally, clinical and safety studies for any potential antivirals need to be conducted soon alongside the development of treatment protocols in pregnant women.
